# Regulation of Phosphatidic Acid Metabolism by Sphingolipids in the Central Nervous System

**DOI:** 10.1155/2011/342576

**Published:** 2010-11-07

**Authors:** Susana J. Pasquaré, Virginia L. Gaveglio, Norma M. Giusto

**Affiliations:** Instituto de Investigaciones Bioquímicas de Bahía Blanca, Universidad Nacional del Sur and Consejo Nacional de Investigaciones Científicas y Técnicas (CONICET), B8000FWB Bahía Blanca, Argentina

## Abstract

This paper explores the way ceramide, sphingosine, ceramide 1-phosphate, and sphingosine 1-phosphate modulate the generation of second lipid messengers from phosphatidic acid in two experimental models of the central nervous system: in vertebrate rod outer segments prepared from dark-adapted retinas as well as in rod outer segments prepared from light-adapted retinas and in rat cerebral cortex synaptosomes under physiological aging conditions. Particular attention is paid to lipid phosphate phosphatase, diacylglycerol lipase, and monoacylglycerol lipase. Based on the findings reported in this paper, it can be concluded that proteins related to phototransduction phenomena are involved in the effects derived from sphingosine 1-phosphate/sphingosine or ceramide 1-phosphate/ceramide and that age-related changes occur in the metabolism of phosphatidic acid from cerebral cortex synaptosomes in the presence of either sphingosine 1-phosphate/sphingosine or ceramide 1-phosphate/ceramide. The present paper demonstrates, in two different models of central nervous system, how sphingolipids influence phosphatidic acid metabolism under different physiological conditions such as light and aging.

## 1. Introduction

Sphingolipids are integral components of eukaryotic cell membranes. There is increasing evidence that sphingolipids are involved in the regulation of various cellular functions such as the action of enzymes and receptors, membrane transport, and signal transduction [1–4] The sphingolipid *de novo* synthesis pathway is an evolutionarily conserved route that generates and interconverts various sphingolipids such as Cer, Sph, C1P, and S1P [5].

**Figure 1:**
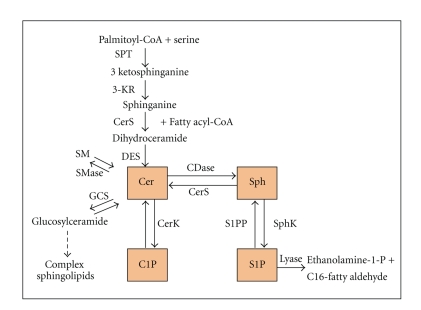
Metabolic pathways of sphingolipid metabolism. Ceramide (Cer) is either sinthesized by *de novo* pathway through the sequential action of *serine palmitoyl transferase *(*SPT*), *ketosphinganine reductase *(*3-KR*), *ceramide synthase *(*CerS*), and *dihydroceramide desaturase* (*DES*), or it is generated from sphingomyelin (SM) hydrolisis by *sphingomyelinase enzyme* (SMase). Cer could be converted into sphingosine (Sph) by *ceramidase* (CDase) action. *Ceramide kinase* (CerK) and *sphingosine kinase* (SphK) generate ceramide 1-phosphate (C1P) and sphingosine 1-phosphate (S1P), respectively.

 Cer is the central molecule in the metabolism of sphingolipids. It is produced via *de novo* biosynthetic pathway beginning with the condensation of serine and palmitoyl-CoA by the enzyme serine palmitoyl-CoA transferase. Cer is also produced by the hydrolysis of sphingomyelin (SM) by sphingomyelinases. It can be either phosphorylated by Cer kinase (Cerk) to C1P or used in the synthesis of SM or glycosphingolipids. Cer can also be broken down by ceramidases to Sph, which, in turn, is phosphorylated by Sph kinases (SphK) to generate S1P. The latter is degraded by specific phosphatases and LPPs that regenerate Sph or by a lyase that cleaves it irreversibly into ethanolamine 1-phosphate and palmitaldehyde [6] (Figure 1). The enzymes involved in sphingolipid metabolism are regulated by physiological and environmental stimuli. Increasing evidence points to a role of this signaling pathway in response to stress, activation of receptors, and pathogenesis [7–9]. Cer is a family of about 50 different molecular species that are characterized by various acyl chains. Highly hydrophobic Cer is generated by membrane-associated enzymes and exerts its effects either in close proximity to the generation site or require specific transport mechanisms to reach its targets in another intracellular compartment [10]. Cer appears to be able to flip-flop across the membrane [11]. However, spontaneous interbilayer transfer is extremely slow [12]. Therefore, the transfer of Cer between intracellular compartments is facilitated by vesicular transport pathways [13] or by a nonvesicular pathway involving a transfer protein from its generation site in the endoplasmic reticulum to the Golgi where it is required for SM synthesis [14]. Cer itself is an important second messenger in various stress responses and growth mechanisms. Its formation occurs in response to many stressinducers [7, 15]. The accumulation of Cer in plasma membranes basically induces significant structural alterations in the membrane bilayer [16]. In addition, Cer has been shown to induce transmembrane translocation of other membrane phospholipid components, ending in the disappearance of their asymmetric distribution [17]. By forming membrane microdomains, Cer favors the activity of certain lipolytic enzymes such as phospholipase A2 [18] it serves to cluster and aggregate activated receptor molecules [19], it regulates the intracellular enzymes such as protein kinase C [20], tyrosine kinases, diacylglycerol kinase, and phospholipases [21], and it alter gene expression [22]. 

C1P is another phosphorylated bioactive sphingolipid whose importance has only recently begun to be appreciated. It is required for the production of prostaglandins in response to several inflammatory agonists. Lamour et al. (2007) demonstrate that Cer kinase localizes in areas where eicosanoid synthesis occurs [23]. Furthermore, C1P has been found to be involved in the stimulation of cell proliferation [24], phagocytosis [25], inflammation [26], and cell survival [27]. The discovery of phosphatases such as lipid phosphate phosphatases (LPPs) that are able to hydrolyze C1P [28] together with the existence of specific Cer kinases [29] suggested that Cer and C1P are physiologically interconvertible. C1P was, in fact, found to inhibit the activation of acid sphingomyelinase and the subsequent generation of Cer [29].

 Sph may also be an important physiological regulator because it can not only inhibit protein kinase C but also induce cell cycle arrest and apoptosis. S1P has different roles in cell growth and survival, angiogenesis, vasculogenesis, neuritogenesis, and immune function. The number of reports on S1P-mediated cell signaling has increased in recent years [30, 31]. Extracellular actions of S1P are mediated by its interaction with a family of five specific G-protein-coupled receptors (GPCRs) known as S1P1-S1P5 [31–35]. In addition, similar to other potent lipid mediators, S1P has further intracellular actions independently of these receptors which are important for the regulation of cellular functions and various kinases [3, 4, 31]

 After summarizing the literature on Cer metabolism, functions, and established signaling pathways in different tissues, our purpose in this paper is to put together and discuss the recently uncovered information that highlights their possible functions in the retina and synaptic endings.

## 2. Sphingolipids and Enzymes Related to Lipid Metabolism

This section summarizes basic knowledge on the modulation of sphingolipids on the enzymes involved in lipid metabolism.

### 2.1. Phospholipase A2 (PLA2)

Phospholipase A2 superfamily consists of a broad range of enzymes characterized by their ability to catalyze the hydrolysis of the middle (sn-2) ester bond of substrate phospholipids. The hydrolysis products of this reaction, free fatty acids, and lysophospholipids, which are derived from the activity of a diverse and growing superfamily of PLA2 enzymes [36], have many important downstream roles. Previous studies have shown that sphingolipid-related compounds such as SM, Cer, C1P, and S1P regulate the activities of PLA2s, including secretory PLA2 (sPLA2) [37, 38] and cytosolic PLA2*α* (cPLA2*α*) [39–42]. A cell-permeable Cer analog was found to increase prostaglandin production, and although Cer alone has little effect, it was found to enhance interleukin-1-stimulated PGE2 production [43]. On the other hand, Cer [36] and calcium ionophore, A23187, were shown to synergize the translocation of cPLA2*α*, but again Cer alone had no effect. Thus, it was suggested that Cer regulates eicosanoid synthesis by enhancing the activation of cPLA2*α*. Furthermore, it has been observed that Cer not only stimulates several phospholipases [44, 45] but also influences sPLA2IIa fatty acid specificity [18, 46]. In contrast to SM, which increases specificity for C20:4 release by preferential inhibition of C18:2 release, Cer appears to directly stimulate the release of C20:4 in preference to C18:2 [37]. These findings show that in the membrane, SM and Cer regulate not only the activity of phospholipases but also the release of C20:4, the precursor of eicosanoids [38]. SM/Cer cycle is stimulated by various hormones, cytokines, and growth factors [47, 48] and plays an important role in inflammatory responses. SM is a physiological inhibitor of several lipolytic enzymes, including sPLA2IIa [49, 50], sPLA2V [49], and cPLA2 [51]. Gesquiere et al. (2002) reported the regulation of sPLA2IIa and sPLA2V by SM and Cer [49]. Koumanov et al. (2002) reported that long-chain Cer not only simulates sPLA2IIa but also promotes the release of polyunsaturated fatty acid from PE/PS substrate [18]. They proposed that polyunsaturated phospholipids are specifically excluded from the Cer-rich lamellar phase, and this results in their increased susceptibility to PLA2 attack. Other reports indicate that C1P rather than Cer functions as the proximal mediator of arachidonic acid (C20:4) release [52]. On the other hand, cell-specific and agonist-dependent events coordinate the translocation of cPLA2*α* to the nuclear envelope, endoplasmic reticulum, and Golgi apparatus [53], thus increasing the enzyme activity [26]. At these membranes, cPLA2*α* hydrolyzes membrane phospholipids to produce C20:4, which initiates pathways leading to eicosanoid synthesis [53]. This phenomenon is triggered by C1P. Recent research supports the idea that C1P allosterically activates cPLA2*α* and enhances the *in vitro* interaction of this enzyme with its membrane substrate phosphatidylcholine (PC). Thus, C1P increases membrane residence time of cPLA2, reminiscent of other interactions of peripheral proteins with phosphatidylinositol and/or diacylglycerol [54, 55]. Gomez-Muñoz and coworkers showed that C1P is a stimulator of DNA synthesis and that it promotes cell division [56]. The same group also demonstrated that C1P blocks apoptosis through the inhibition of acid sphingomyelinase in macrophages [57]. Furthermore, other groups have shown that C1P is a mediator of phagocytosis by promoting phagosome formation [25], and it has also been demonstrated that Cer kinase and C1P are required for the activation of the degranulation process in mast cells [58]. 

 Among the recognized PLA2s, there is one that does not require Ca^2+^ for activity and is classified as Group VIA iPLA2 and is designated as the *β*-isoform of iPLA2 (iPLA2*β*) [59]. The iPLA2*β* enzyme is involved in phospholipid remodeling and signal transduction [60], and it contributes to apoptosis in many cell types [61–63] by a mechanism that has not yet been elucidated. Recent studies indicate that iPLA2*β* participates in endoplasmic reticulum (ER) stress-induced INS-1 insulinoma cell apoptosis [61, 62]. Furthermore, iPLA2*β* activation and subsequent Cer generation are key components in the crosstalk between ER and mitochondria following induction of ER stress and their involvement serves to amplify ER stress-induced apoptosis of insulin-secreting cells [64]. ER stress leads to iPLA2*β* association with mitochondria, and to Cer generation in both ER and mitochondrial fractions. 

### 2.2. Phospholipase D (PLD) and Lipid Phosphate Phosphatase (LPPs)

PLD hydrolyzes PC in order to produce PA and choline [65, 66]. PA is a biologically active molecule and can be hydrolyzed by lipid phosphate phosphatases to yield diacylglycerol (DAG) [67]. PLD hydrolytic activity can be triggered by a wide variety of agonists such as hormones, neurotransmitters, and growth factors [68–70]. The use of primary alcohols decreases PA production catalyzed by PLD and eventually disrupts a number of cell events. Based on the transphosphatidylation reaction, PA has been shown to function as an important lipid second messenger in a wide variety of cells, such as membrane trafficking, endocytosis, exocytosis, cell growth, differentiation, and actin cytoskeleton reorganization [65, 66]. Lipid phosphate monoesters, including PA, lysophosphatidic acid (LPA), S1P, and C1P, are dephosphorylated by LPPs. They are intermediaries in phospho- and sphingolipid biosynthesis and they also play important roles in intra- and extracellular signaling. The dephosphorylation of these lipids eliminates their signaling activity and generates products with additional biological activities or metabolic fates [67]. LPPs isoforms, termed LPP1 (PAP2a), LPP2 (PAP2c), and LPP3 (PAP2b), have been cloned and are distributed between endomembrane compartments and the plasma membrane.

 Cer has been proposed to inhibit PLD activity [71, 72] by preventing its activation by protein kinases C (PKCs) and monomeric G-proteins [73], by downregulating PLD gene transcription [74] or by a direct effect on the catalytic core of the enzyme [74]. Furthermore, PLD is stimulated by PA and LPA, and this stimulation is abolished by Cer. The mitogenic effects produced by PLD are inhibited by C2- and C6-Cer. In contrast, Sph has the opposite effect to Cer on DNA synthesis. S1P stimulates PLD activity in Swiss 3T3 fibroblasts and C2- and C6-Cer block this activation [75]. Furthermore, cell-permeable Cer inhibits PA-induced [71] and S1P-induced production of PA by PLD. These results indicate that PLD pathway is an important target for the modulation of signal transduction by sphingolipids.

 The specific activity of LPP is increased when Cer is generated by exogenous sphingomyelinase. Therefore, the increased rate of degradation of PA and LPA could be important steps in the termination of the mitogenic signal of these phospholipids, thus inhibiting cell proliferation. Cer can inhibit some effects of S1P by stimulating its degradation via LPP, which also hydrolyzes PA [71]. It has been reported that Sph (i) inhibits Mg^2+^-dependent phosphatidate phosphohydrolase and LPP activities [76, 77], (ii) activates PLD [78], and (iii) stimulates an 80-kDa DAG kinase [79]. These combined actions increase the accumulation of PA relative to DAG [80], which could also decrease protein kinase C activation. It has been demonstrated that the cells with short-chain Cer enhance the dephosphorylation of both PA [71] and S1P. Furthermore, S1P inhibits PA hydrolysis in homogenates of Rat2 fibroblasts. The effects of cell-permeable Cer on the stimulation of the hydrolysis of exogenous PA and S1P by activating a common phosphohydrolase, mitigate the mitogenic activity of these bioactive phospholipids. C2-Cer not only destroys S1P signal but also potentiates an antagonistic one by increasing Cer production from S1P and endogenous sphingolipids. We also must take in account that short-chain ceramides are also hydrolyzed and converted back to long-chain ceramides thus increasing long-chain ceramide availability. LPPs are partly expressed as ectoenzymes on the cell surface [81]. LPPs exert their influence on the physiological responses mediated by lipid phosphates such as S1P or LPA by regulating the availability of the extracellular ligand and also by controlling the accumulation of bioactive lipid phosphates downstream of G-protein receptor activation [81]. LPPs could hydrolyze S1P [82–84], which could, in turn, facilitate the rapid uptake of Sph. Recent studies show that changing the expression of different LPPs modulates S1P-mediated activation of extracellular signal-regulated kinases, PLD, DNA synthesis, cell migration, changes in [Ca^2+^]_*i*_, I*κ*B phosphorylation, and translocation of NF-*κ*B to the nucleus from the cytoplasm and interleukin-8 secretion [81, 85–87]. Sigal and coworkers (2005) [88] showed that increasing LPP activity enhances the uptake of DAG by cells treated with exogenous PA. Thus, LPPs convert lipid phosphates which have very limited ability to enter cells into products that more readily traverse the plasma membrane and which can then signal directly or after phosphorylation. The overexpression of LPP-1 increases the accumulation of intracellular S1P in response to exogenous S1P because of the ectoactivity of LPP-1 [89]. Thus, LPPs can modify the balance of signaling by S1P through three different mechanisms. Firstly, they can decrease extracellular S1P concentrations, thus lowering the activation of cell surface receptors. Secondly, they have been shown to attenuate signaling downstream of the activation of surface S1P receptors. Thirdly, by promoting the formation of intracellular S1P, they increase intracellular signaling by this agonist. These combined observations add to our understanding of the complex interplay between the roles of S1P as an extracellular versus intracellular signaling molecule.

 The above-summarized observations are indicative of the association between Cer and its derived sphingolipids with the enzymes that control PA metabolism. The principal effects exerted by sphingolipids on PLA2, PLD, and LPPs are summarized in Table 1.

## 3. Role of Sphingolipids on PA Metabolism in ROS: Effect of Light

### 3.1. Lipid Phosphate Phosphatases

 Lipid phosphate monoesters, including PA, LPA, S1P, and C1P, are intermediaries in phospho- and sphingolipid biosynthesis and they also play important roles in intra- and extracellular signaling. The dephosphorylation of these lipids terminates their signaling actions and generates products with additional biological activities or metabolic fates [67]. As it was described in the preceding section, the key enzymes responsible for the dephosphorylation of these lipid phosphate substrates are termed LPPs. They display isoforms and cell specific localization patterns which are distributed between endomembrane compartments and the plasma membrane. The role of LPPs in intracellular lipid metabolism and in the regulation of both intra- and extracellular signaling pathways that control different cellular functions has been analyzed in detail [90–93].

ROS are specialized light-sensitive organelles in vertebrate photoreceptor cells. When light is absorbed in the photoreceptor, it causes rhodopsin isomerization, initiating visual excitation. Activated rhodopsin interacts with transducin (T). T*α*GTP activates cyclic GMP phosphodiesterase (PDE), diminishing free cGMP concentration and consequently affecting sodium channel closing. The hydrolysis of T*α*GTP yields the inactive PDE. The cycle is closed because rhodopsin is phosphorylated by rhodopsin kinase and arrestin [94]. Lipids in rod outer segments are of considerable importance not only in providing an adequate environment for efficient phototransduction but also in originating the second messengers involved in signal transduction. ROS have the ability to adapt the sensitivity and speed of their responses to ever changing conditions of ambient illumination. Recent evidence has demonstrated that a major contributor to this adaptation is the light-driven translocation of key signaling proteins into and out of ROS, which constitute the cellular place where phototransduction occurs [95]. It has also been reported that transducin, arrestin and recoverin [96–98] are proteins involved in this mechanism. Previous studies revealed the presence of LPPs and their regulation in isolated ROS from bovine retina [99–102]. It has also been extensively reported that the activity of enzymes involved in ROS phospholipid turnover such as phospholipase C [94, 103], PLA2 [104], phosphatidylethanolamine N-methyltransferase [102], DAG kinase [105], PAP2 [101], phosphoinositide-3-kinase [106, 107], and PLD [108] is modulated by light. 

 In this section, we describe the effects of Cer, Sph, C1P, or S1P on LPPs, and DAGL activities using three different ROS populations: (i) DROS obtained from dark-adapted retinas and purified under dim red light, (ii) LROS obtained from DROS and exposed to room light for the enzyme assays, and (iii) BLROS obtained from light adapted retinas and purified under room light. 

 Three mammalian LPP isoforms termed LPP1 (PAP2a), LPP2 (PAP2c), and LPP3 (PAP2b) have been cloned. In general, LPP1, LPP2, and LPP3 show the major catalytic efficiency to LPA, PA, and S1P, respectively [109, 110], thus altering the balance of bioactive lipid mediators [81, 111]. PA and its dephosphorylated product, DAG, have important functions in signaling and PA itself emerges as a regulator of pleiotropic signaling responses [112]. In our study, it was observed that LPP activities are strongly inhibited in BLROS although no differences in LPP3 levels between DROS and BLROS were found [113]. This is indicative of the involvement of a bleaching process in LPP modulation which could be related either to the absence or to the presence of a specific protein affected by light-driven translocation. These findings agree with our previous observations which demonstrated that light inhibition of LPP activity in ROS is a transducin-mediated mechanism [101]. PLD is inhibited by light as it occurs with LPPs [108]. On the other hand, it has been reported that diacylglyceride kinase (DAG kinase) [105] is modulated by light in the opposite manner as it occurs with LPPs and PLD. This could be indicative of the fact that PA and DAG levels have physiological relevance in ROS under illumination; that is, under light conditions, an increased DAG kinase activity promotes a higher PA availability whereas under dark conditions an increase in PLD/PAP activities yields a higher DAG availability [101, 108].

 It was observed that the major inhibitory effect on PA hydrolysis is exerted by S1P in DROS and by C1P in BLROS. Furthermore, C1P was found not to modify LPPs activities from LROS whereas in the presence of S1P, LPPs activities were found to be stimulated [113]. The results observed in the presence of S1P are indicative of the presence of LPP3 in ROS. This isoform has been localized with PLD in caveolin-enriched detergent-resistant microdomains where it metabolizes phospholipase D2-derived PA [114, 115]. C1P is a potent inhibitor of protein phosphatases (PP) which have been found to be involved in the inhibition of LPPs in isolated ROS [116–118]. C1P also seems to exert a direct action on LPPs. In this respect, it has been reported that C1P is required for the activation and translocation of other enzymes involved in lipid metabolism such as cPLA2 [30]. In order to evaluate the effect of peripheral and soluble protein depletion on LPPs activities, enzyme assays were carried out in DROS membranes and BLROS were washed with low ionic strength buffer. The same LPPs activities and similar dark/light differences were observed both in ROS and in depleted membranes. In the presence of either S1P or C1P, DAG generation from PA in depleted DROS and BLROS was lower than the activity determined in the presence of PA alone. In order to determine if the effect of S1P and C1P on DAG production is due either to their competitive characteristics and/or to Sph and Cer, the respective dephosphorylation products of S1P or C1P by LPPs, Sph, or Cer were included in PA hydrolysis assays. In entire DROS and BLROS, Sph and Cer were found to inhibit DAG production in a similar percentage. In DROS and BLROS depleted of soluble and peripheral proteins, Sph and Cer were found to inhibit DAG formation to a major extent [113]. These results suggest not only a competitive effect between PA and S1P or C1P but also a direct effect of Sph and Cer on LPPs. In this respect, it has been reported that Sph not only inhibits DAG formation but also stimulates PA formation, thus inhibiting LPPs and stimulating PLD and DAGK [119, 120]. Increased intracellular Cer levels have been involved in the activation of photoreceptor apoptosis [121]. Analyses of Drosophila phototransduction have indicated that Cer-kinase-mediated maintenance of Cer level is important for the local regulation of PIP2 and PLC during phototransduction [122]. Furthermore, it has been suggested that LPP2 and LPP3 play an important role in apoptotic processes. This is supported by the fact that DAG and Sph, the products of LPPs, are involved in apoptosis induction [111], while S1P has antiapoptotic roles [123].

### 3.2. Diacylglycerol Lipase

DAGL is coupled to LPP and these enzymes appear to work as in an enzymatic complex. DAG generation, and its partial degradation to MAG by DAGL is a phenomenon that has been extensively analyzed in our laboratory [100] on the premise that DAG produced from PA for LPP activity is metabolized to MAG [99–101, 118]. The metabolism of DAG generated from PA by LPP activity in ROS was evaluated under the same experimental conditions specified by LPPs. MAG, the product of DAGL, was evaluated in the presence of either Cer, Sph, C1P, or S1P. Both DAGL and LPPs were inhibited in a similar manner by light [124, 125]. Low concentrations of S1P and C1P were found to maximally inhibit MAG production in DROS. MAG formation was observed to be inhibited by C1P in BLROS and LROS. The effects of Cer and Sph on DAGL activity were also analyzed. It was observed that in DROS, Sph and Cer inhibited MAG whereas in BLROS, they both stimulated MAG formation [124]. 

 Similary to what occurred with LPPs, in depleted DROS, DAGL activity was inhibited with respect to entire DROS, reaching similar values to those in LROS [113, 124]. In depleted DROS, MAG generation was inhibited in the presence of S1P and C1P. In depleted BLROS, on the other hand, MAG production was found in a higher percentage in the presence of C1P, showing no significant differences in the presence of S1P. In depleted DROS and BLROS, Sph and Cer were found to stimulate MAG formation. 

 DAG has unique functions as a basic component of membranes, as an intermediary in lipid metabolism and as a key element in lipid-mediated signaling. In addition to PKC family, an increasing number of proteins are known to be modulated by DAG [126, 127]. It was observed that in excised patches from frog rod outer segments, dioctanoylglycerol (DiC8) modulates the gating of the cGMP-gated channel in the absence of a phosphorylation reaction [128]. There are three possible pathways of MAG formation using PA as substrate: (i) by the action of LPP/DAGL, (ii) by the action of PLA/LPP, and (iii) by the action of PLA/LPA lysophosphatase. Our studies demonstrated that LPP/DAGL is the pathway operative in ROS [113, 124]. The functional significance of light modulation in DAGL activity in vertebrate photoreceptors has not been fully elucidated to date. However, evidence of the role of DAGL in Drosophila phototransduction has been reported [129, 130]. Under all conditions assayed, DAGL substrate (DAG) was found to be diminished in the presence of S1P [113] whereas MAG production was observed to be inhibited in entire and depleted DROS while it underwent no changes in entire and depleted BLROS [113]. Summing up, DAGL activity was found to be inhibited in the presence of S1P in entire DROS and stimulated in depleted DROS entire and depleted BLROS. The fact that S1P diminishes DAGL activity in DROS and that it produces a stimulatory effect on DAGL in ROS membranes where protein redistribution occurs (BLROS), or where soluble or peripheral proteins are detached (depleted DROS), seems to indicate that S1P produces its effect either by modulating or interacting with a protein involved in the phototransduction cascade that modulates DAGL activity.

 Interestingly, no LROS/BLROS differences were observed in DAGL activity in the presence of C1P or S1P [124]. These findings were not observed in LPP [113], thus suggesting that they are related to DAGL itself and that the high inhibition observed in DAGL activity caused by bleaching is partially compensated by S1P or C1P. Furthermore, Sph or Cer generated from S1P and C1P by LPP may modify DAGL activity, as corroborated by our observations of the effect of Sph and Cer on MAG generation in entire DROS. The fact that S1P and/or C1P in depleted DROS and in entire and depleted BLROS have the opposite effect to Sph and Cer suggests that these lipids act independently on the enzymatic activity. DAGL from DROS was observed to decrease in the presence of Cer, reaching similar values to those of BLROS. Further observations indicate that Cer seems to induce either protein migration or detachment of DAGL enzyme from the ROS membrane to the cytosolic fraction. The cellular ratio between S1P/Sph and C1P/Cer is a critical factor in cell survival/cell death decisions. It has been reported that DAG, Cer [121] and Sph are involved in apoptosis induction [111] whereas S1P has an antiapoptotic role [123]. The concentration of DAG in small membrane areas, its characteristic negative curvature, and its lack of charge induce unstable, asymmetric regions in membrane bilayers. Intermediaries with increased curvature minimize this tension and are essential for membrane fusion and fission processes [131]. Consequently, DAG may affect physiological processes by altering the membrane structures and fluidity and may favor the shedding of membranous disks. 

**Figure 2:**
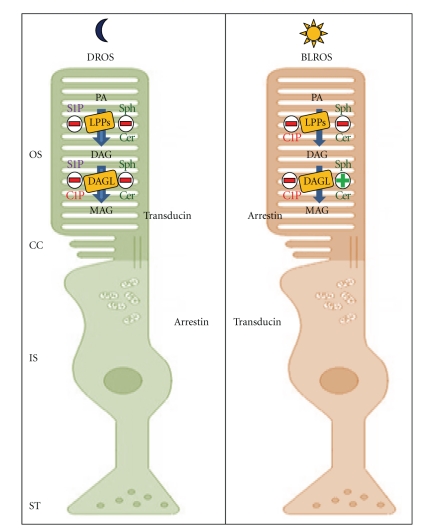
Modulation of lipid enzymatic activities by sphingolipids in two experimental models of isolated rod outer segments from vertebrate retinas under dark (DROS) and light (BLROS) conditions. Under dark condition Cer, Sph, and their phosphorylated products, S1P and C1P, diminish LPPs and DAGL activities. Under light condition, both Sph and Cer stimulate DAGL activity. These effects depend on the presence of soluble and peripherial proteins, as was observed in depleted DROS and BLROS where LPPs' inhibition produced by sphingolipids is higher than in entire ROS. Interestingly, in depleted DROS, the absence of these proteins produces an increase in DAGL activity. These results indicate that protein translocation (transducin and arrestin) between inner and outer segment or protein activation, caused by light exposure, could modulate enzymatic activities involved in PA metabolism. The relative size of arrows indicates the different degree of PA metabolism in DROS and BLROS.

In the light of these findings on LPP and DAGL, it can be concluded that the pathway involving LPP/DAGL has an important role in controling PA/DAG/MAG levels. Taken together, the above-mentioned results also suggest that the metabolism of PA/DAG/MAG following light-mediated ROS stimulation plays an active role in the organization of signaling responses following the initial light stimulus and that Cer and its derivates have an important role in the light effects observed. We, therefore, suggest that the proteins related to phototransduction phenomena are involved in the effects observed in the presence of either S1P/Sph or C1P/Cer. The main findings about the role of Cer and sphingolipids derived from it in ROS under dark and light conditions are summarized in Figure 2.

## 4. Role of Sphingolipids on PA Metabolism in Cerebral Cortex Synaptosomes: Effect of Aging

### 4.1. Sphingolipids, Aging and Neurodegenerative Diseases

Alterations in glycerophospholipids, sphingolipids, and cholesterol have been reported to occur in aging, neurodegenerative process and various neurological disorders [132–135]. Higher levels of cholesterol and of lipid mediators derived from glycerophospholipids and sphingolipids were, in fact, found to be significantly increased in these disorders [136, 137]. 

 Lipid mediators from sphingolipid metabolism, namely Cer, C1P, Sph, and S1P, modulate cPLA2, sPLA2, and cyclooxygenase-2 activities through the translocation of these enzymes from the cytosol to nuclear and plasma membranes [37, 38, 138, 139]. The accumulation of lipid mediators derived from glycerophospholipids and sphingolipids, along with changes in the cellular redox status, and the lack of energy generation are associated with neural cell injury and cell death and neurodegenerative diseases [2, 138, 140, 141]. Sphingolipids, including Cer and Sph, accumulate in several tissues such as brain during aging [142, 143]. 

 Several lines of evidence support the postulation that age-related neurodegenerative diseases such as Alzheimer's disease (AD) are related to sphingolipid metabolism [144–146]. Previous research indicates, in fact, that Cer significantly accumulates in the brain of AD patients [147]. Thus, Cer/Sph accumulation occurs with development and aging, and it plays important roles in regulating cell proliferation, differentiation, and apoptosis. 

 Although Cer generation in cells is usually associated with the promotion of apoptosis, in some cell types including sympathetic neurons, Cer promotes cell survival [148]. Gomez-Munoz's reports indicate that C1P and Cer are antagonistic signals and that C1P blocks cell death by inhibiting Cer production [57, 149]. Cer is a potent inhibitor of protein kinase B (PKB), which is downstream of phosphatidylinositol 3-kinase, and this is part of the proapoptotic effect of Cer [149]. Therefore, depletion of Cer seems to facilitate PKB activation. A decrease in SM and an accumulation of Cer have both been found to be involved in AD [147]. A high Sph level was also found in AD brains although S1P levels were low. Both the low levels of S1P and the high levels of Cer in AD brains seem to contribute to the disease pathogenesis. *In vitro* amiloyd *β* (A*β*) has been shown to induce apoptosis via SM/Cer pathway in brain [150, 151]. 

 Cer levels were also found to increase in response to aging and various age-related stress factors (e.g., oxidative stress) and were directly involved in apoptotic signaling in various cell types, including neurons [147, 152–154]. It has thus been suggested that Cer and A*β* synergize to induce neuronal death in AD. Using *in vitro* and* in vivo* models of AD, it has been shown that A*β* in neurons [146, 147] and oligodendrocytes [155] increase Cer levels [156, 157]. Further studies have demonstrated that Cer levels increase A*β* synthesis [144, 158] and favor gamma secretase processing of APP [157, 159, 160] so that inhibition of Cer synthesis confers protection against A*β* [147]. Satoi et al. (2005) found that Cer levels were also increased in the cerebrospinal fluid of AD patients [161]. He et al. (2010) recently reported the first evidence of acid sphingomyelinase activation, Sph increase, and S1P decrease [162]. Aging results in the accumulation of various stress factors, including proinflammatory and oxidative stress molecules that can stimulate sphingomyelinase activities, leading to the production of the proapoptotic lipid, Cer [147, 163]. Thus, under normal circumstances, A*β* and Cer levels may be balanced to maintain neuronal cell homeostasis, but upon aging, various stress factors become elevated to the extent that they may activate sphingomyelinases and produce Cer. Accumulating evidence also supports a role of Sph in apoptosis [164–166]. 

 In contrast to Cer and Sph, S1P can enhance cell proliferation and antagonize apoptosis [167]. The regulation of sphingomyelinases and ceramidases, as well as Sph kinase, S1P phosphatase and LPPs may play pivotal roles in the apoptotic signaling of cells by regulating the ratio between SM, Cer, Sph, C1P, and S1P. Stress signals lead to increased levels of Cer [153, 168]; however, the association of Cer with downstream signaling events is still poorly understood. Cer regulates directly or indirectly the activities of different cell signaling mediators including Cer-activated protein kinases and Cer-activated protein phosphatase, mitogen-activated protein kinase, protein kinase *ζ*, phospholipases such as cPLA, PLD, and PLA2, stress-activated protein kinases, cyclo-oxygenase, transcription factors such as the nuclear factor *κ*B, and caspases [168–174]. 

 The major Cer species in the brain are C18:0-, C18:1-, and C24:1-Cer. All Cer species were found to be elevated in ischemic brain and Cer intracellular site accumulation was observed to occur in purified mitochondria as well as in the endoplasmic reticulum [175]. Futhermore, it has been reported that ethanol induces Cer formation in astrocytes and that PA, the product of PLD activity, antagonizes ethanol-induced formation of Cer. These results evidence a crosstalk between PA and Cer, two lipid messengers with opposite effects on cellular proliferation. It is also known that PA mediates mitogenic stimulation in astrocytes whereas the formation of Cer by sphingomyelinase activation accompanies apoptosis. Summing up, these findings are indicative of a crosstalk between lipid-signaling pathways in astrocytes such that the product of PLD, namely PA, inhibits Cer formation whereas Cer inhibits PLD activation. PA:Cer ratio contributes to the decision whether astrocytes proliferate or undergo apoptosis [176].

### 4.2. Sphingolipids, PA Metabolism and Aging

Aging is accompanied by the impaired functioning of many systems, thus producing a gradual decline in the capacity of various cell types including neurons [177]. Lipids have broad information carrying functions in the central nervous system. They form an integral part of membranes and provide messenger molecules that mediate communication among cells. Any modification in their metabolism and/or in the enzymatic activities that metabolize them may, therefore, affect cell function in physiological aging. Age-related changes in lipid content and in the enzymatic activities involved in lipid metabolism in different brain regions have been documented [142, 178–185]. PA, DAG, and 2-AG are involved in signal transduction [136, 186, 187]. In eukariotic cells, these molecules have been associated with neurological disorders such as AD [188]. 

 Previous research from our laboratory demonstrated that LPP hydrolyzes PA in synaptosomal cerebral cortex and that the generated DAG is metabolized to MAG by DAGL [181]. LPP regulates cell signaling under physiological or pathological conditions. This cell signaling occurs via the attenuation of lipid phosphate signaling and the production of bioactive DAG, 2-AG, Sph, and Cer [81]. The precise control of PA, DAG, and 2-AG and the enzymes that metabolize them, LPP, DAGL, and MAGL are necessary for the correct functioning of these molecules in the signaling mechanism. 

**Figure 3:**
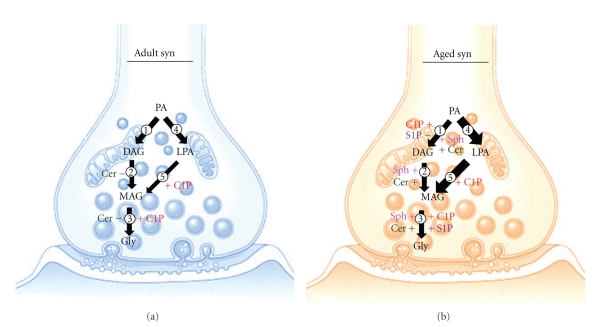
Modulation of PA metabolic pathways by sphingolipids in synaptosomes (Syn) from adult (a) and aged (b) rat cerebral cortex. In adult Syn (a), Cer modulates DAGL and MAGL activities negatively while their phosphorylated form, C1P, stimulates MAG and Gly production via LPAPase/MAGL pathway. In aged Syn (b), results indicate that there is a major modulation by sphingolipids. Cer and Sph stimulate PA metabolization via LPPs/DAGL/MAGL pathway. C1P increases LPAPase, DAGL, and MAGL activities. In contrast, S1P inhibits DAG generation but produces an increase in Gly formation. (1) LPPs, lipid phosphate phosphatase; (2) DAGL, diacylglycerolipase; (3) MAGL, monoacylglycerolipase; (4) PLA, phospholipase A; (5) LPAPase, lysophospholipase phosphatase or LPPs. The relative size of arrows indicates the predominance of PA metabolism pathway.

 The present paper also analyzes the formation of lipid mediators generated from PA in synaptosomes prepared from the cerebral cortex of adult and aged rats. In all instances, PA metabolism was analyzed in the presence of the sphingolipids. Our results demonstrate that aging modulates PA metabolism and indicate a different utilization of PA in the presence of S1P and C1P [125]. On the other hand, PA metabolism was found to generate DAG, MAG, and glycerol by the sequential action of LPP, DAGL, and MAGL in the CC Syn (Figure 3). It was also demonstrated that PA is metabolized by PLA/LPAPase in synaptic endings. In adult CC Syn, DAG formation was found to be stimulated at low concentrations of C1P and MAG and glycerol generation was lower in aged than in adult CC Syn. Equimolar concentrations (100 *μ*M) of C1P and PA were observed to generate a DAG level similar to that observed with PA alone and a higher production of MAG and glycerol in aged CC syn with respect to adult ones. DAG production was found to be inhibited as a function of S1P concentration in aged CC synaptosomes, and no changes in DAG production by S1P were observed in adult CC syn. It was also observed that MAG formation in adult and aged CC syn underwent no changes in the presence of S1P; however, glycerol production was higher in adult than in aged membranes in the presence of increased S1P concentrations. We also evaluated the effect of Sph and Cer on these products in CC Syn from adult and aged rats. Sph and Cer produce no changes in DAG production in adult membranes. However, in aged membranes, DAG production is stimulated by both Sph and Cer. Sph was found to produce no changes in MAG production in adult Syn but stimulated it in aged CC Syn. Cer, in contrast, was found to inhibit MAG generation in adult membranes and stimulated its formation in aged membranes. Furthermore, in the presence of Cer, glycerol formation was found to be inhibited in adult membranes but stimulated in aged membranes [125]. 

The transformation of DAG into MAG plus glycerol in Syn preincubated with RHC-80267, a specific DAGL inhibitor, allowed us to better visualize the differences in DAG metabolism between adult and aged CC synaptosomes. A major transformation of DAG into MAG and glycerol was observed in adult Syn. In the presence of C1P, DAG transformation into MAG and glycerol was observed to be markedly lower in aged membranes with respect to adult membranes. No further significant differences were observed in DAG metabolism between adult and aged membranes in the presence of S1P. DAG metabolism in the presence of Cer and Sph was also evaluated. In the presence of Cer and Sph, DAG was found to be transformed into MAG and glycerol mainly in adult membranes [125].

 PA [112], DAG [189], 2-AG [190], Sph, Cer, and their phosphorylated products [30, 191] have been defined as key inter- and intracellular lipid signaling molecules. All of them and their related enzymes participate in the regulation of many functions of the CNS [192, 193]. PA metabolism in CC syn involves two possible pathways: (i) the sequential action of LPPs, DAGL, and MAGL [183, 184] generating DAG, MAG, and glycerol, respectively, and (ii) the action of PLA, LPA phosphohydrolase, and MAGL generating LPA, MAG, and glycerol, respectively (Figure 3). The fact that the product of an enzymatic reaction could be used as a substrate for the subsequent enzyme indicates that these enzymes behave as an enzymatic complex. A sequential action of LPP and DAG lipase producing DAG and MAG, respectively, was observed in rat CC Syn [184]. It has been reported that endogenously produced DAG as a result of LPP action is further hydrolyzed to MAG and glycerol [100, 194]. C1P, S1P, Cer, and Sph are found in Syn at negligible concentrations. In general, tracer lipids such as S1P are present at low nanomolar concentrations in cells but at high nanomolar concentrations in serum [195]. Cer often constitutes 0.1%–1% of total membrane lipids and Sph is often detected at concentrations that are lower than an order of magnitude than those of Cer [196]. The degree of competitiveness observed between PA/S1P suggests that LPP1 is the most active isoform in adult Syn, while LPP1 and LPP3 isoforms are most active in aged Syn. As a result, LPP1 in adult synaptosomes and LPP1/LPP3 in aged Syn could either restrict the effects of S1P to their respective receptors and/or participate in their uptake, thus exerting their influence on synaptosomal functions [125, 177]. DAG generation is quantitatively different from that observed in MAG and glycerol formation in the presence of C1P and S1P, thus suggesting that these effects on MAG and glycerol are related to DAGL/MAGL or PLA/LPA phospholipase/MAGL themselves and that they are not a consequence of different degrees of DAG availability. Under our assay conditions, it could be observed that MAG and glycerol from PA involve two possible routes: (i) the sequential action of LPP/DAGL/MAGL and (ii) PLA/LPA phosphohydrolase/MAGL [183]. The use of RHC-80267, a specific DAGL inhibitor [197], enabled us to determine the different pathways involved in MAG and glycerol production. In adult CC synaptosomes, the two pathways contribute equally to MAG generation whereas in aged CC synaptosomes the second pathway is predominant (Figure 3). Though MAG availability is similar in adult and aged Syn, its metabolism to glycerol is lower in aged membranes. This seems to indicate that aging diminishes the catalytic efficiency of MAGL for its substrate. When the DAGL pathway is inhibited, glycerol production decreases only in aged Syn, thus corroborating the above-mentioned hypothesis. It was also observed that under lower availability of the substrate, MAGL becomes more sensitive to the presence of C1P and S1P, reducing the activity in adult Syn and increasing it in aged Syn. The analysis of the metabolism of DAG to MAG plus glycerol by DAGL/MAGL indicated that aging reduces this metabolism whereas in the presence of C1P or S1P, metabolism undergoes no changes. Based on our results, it can be hypothesized that C1P and S1P modulate MAG and glycerol formation by the pathway involving PLA/LPAPase/MAGL. In view of the above, and in order to assess whether or not the effect of S1P and C1P on DAG, MAG, and glycerol production is due to the Sph and Cer generated by LPP on S1P or C1P, we evaluated the effect of the two lipids on these products. Our results demonstrate that S1P and Sph have different effects on glycerol generation from adult Syn and on DAG and MAG generated from aged membranes. In addition, C1P and Cer exert different effects on DAG/MAG/glycerol in adult membranes. The different effects of S1P and C1P on PA metabolism with respect to those of Sph and Cer suggest that these lipids modulate the enzymatic activities that metabolize PA by independent mechanisms. S1P exerts its effects mainly through related membrane receptors [198, 199] whereas PA, DAG, C1P, and Cer do so by the recruitment of cytosolic proteins [200]. 

 Summing up, this paper section analyzes the aging effect on PA metabolism by means of the sequential action of LPPs/DAGL/MAGL and the pathway involving PLA/LPAPase. The analysis of DAG production using PA and S1P or C1P at equimolar concentrations shows the competitive effect between PA and these alternative substrates. However, the effects of the alternative substrates at concentrations other than equimolar with PA on MAG and glycerol production may be due not only to different DAG availability but also to the effect of the alternative substrate itself on the enzymes that subsequently metabolize DAG or generate MAG and glycerol. Figure 3 summarizes the principal findings observed in adult and agedSyn. 

 Recent advances in neuroscience have demonstrated that lipids have extensive information-carrying functions in the central nervous system both as ligands and as substrates for proteins [201]. PA and PA-lipid derivatives mediate a diverse range of biological processes in the CNS. On the other hand, PA and DAG alter membrane properties, control traffic, and serve as messenger molecules mediating communication among cells [202]. MAG functions as an endogenous CB-1 receptor agonist [190]. An imbalance of PA, DAG, or 2-AG may induce alterations both in neurotransmission and in the neuronal dysfunction observed in senescense and in neurological disorders such as Parkinson and AD, demonstrating the crucial role of lipids in tissue pathophysiology and cell signaling [203, 204]. Signaling lipid-generating enzymes from PA may thus provide pharmacologically potential targets for the treatment of aging and neurological dysfunctions. 

 Sphingolipid signaling may also represent a novel neuroprotective target to counteract the pathophysiology of acute brain and spinal cord injury at the level of apoptotic cell death mechanisms, mitochondrial dysfunction, lipid hydrolysis, and oxidative damage mechanisms. Furthermore, S1P acting as an agonist seems to increase CNS resistance to injury by promoting neurotrophic activity and antagonists of certain S1P-related activity are likely to have proregenerative effects via the promotion of neurite growth [205]. Recent findings suggest possible roles of S1P in regulating apoptotic cell death, oxidative stress and damage mechanisms, mitochondrial dysfunction, and modulation of trophic factor responses, including neurite outgrowth and neuro- and angiogenesis [206, 207]. Another reason why S1P is involved in acute CNS injury is the fact that sphingolipid signaling is known to play a role in membrane lipid hydrolysis, which has long been known to be one of the earliest events in the posttraumatic secondary injury cascade. Membrane lipid molecules seem to play an important regulatory role as signaling molecules and second messengers, which under pathological conditions can undergo oxidative damage in the form of lipid peroxidation [208]. A third potential linkage between sphingolipid signaling and CNS injury is derived from the known importance of reactive oxygen species (ROS) and oxidative damage mechanisms in the pathophysiology of CNS injuries [208–210]. Recently, a crosstalk has been proposed between metabolites of glycerophospholipid and sphingolipid metabolism, which is an important step in the initiation and maintenance of oxidative stress associated with neurologic disorders [140, 208]. Oxidative stress has been found to be involved in the pathogenesis of traumatic brain and spinal cord injuries. 

 Further research in this novel area will, therefore, lead to a better understanding of the mechanisms controling glycerophospholipid and sphingolipid metabolism in the CNS and will provide potential targets for diagnostic or therapeutic strategies for the treatment of aging and neurological diseases.

## 5. Summary and Concluding Remarks 

There is a close relationship between glycerophospholipid and sphingolipid metabolism. Alterations in both of them have been reported to occur in invertebrate phototransduction, aging, neurodegenerative processes, and various neurological disorders. In this paper, we have summarized the principal findings that relate PA metabolism with Cer, Sph, C1P, and S1P in vertebrate phototransduction and in aging phenomena. PA is metabolized by LPPs which also dephosphorylate S1P and C1P. The product of LPPs action on PA, DAG, is additionally metabolized to MAG in ROS and to MAG and glycerol in CC Syn. DAGL and MAGL enzymes participate in these degradative mechanisms, and they, therefore, seem to work similarly to an enzymatic complex. In ROS, the principal competitive effect on PA hydrolysis is exerted by S1P in darkness and by C1P, when retinas are bleached and both sphingolipids reduce DAGL activity under dark condition. Furthermore, S1P and C1P decrease MAG production under dark conditions whereas only C1P diminishes its formation in bleached ROS. Sph and Cer inhibit DAG and MAG formation in entire ROS independently of the their illumination state. The extraction of peripheral and soluble proteins from ROS promotes the metabolism of DAG to MAG under stimulation by light. We, therefore, suggest that proteins related to phototransduction phenomena are involved in the effects observed in PA/DAG/MAG metabolism in the presence of either S1P/Sph or C1P/Cer. However, it cannot be disregarded that high levels of lipid mediators could modulate calcium homeostasis and that this may be, in part, responsible for the effects observed on LPPs and DAGL activities under light conditions. 

 On the other hand, changes in PA metabolism in CC Syn have been observed in our model of aging in the presence of Cer, Sph, S1P, and C1P. Opposite effects have also been observed between S1P and C1P on PA metabolism in aging, while S1P decreases DAG formation, C1P increases it and favors its metabolism to MAG. The dephosphorylated products of C1P and S1P, Cer, and Sph increase PA metabolism by the pathway involving LPP/DAGL/MAGL action in aged membranes. Available evidence emphasizes the key role of sphingolipid molecules and their relative balance in regulating the final fate of photoreceptors and degenerative process in the CNS. The involvement of these molecules in the modulation of enzymes that generate second lipid messengers from glycerophospholipids open an important research pathway. Therefore, further studies in this direction will lead to a better understanding of the mechanisms controling PA metabolism in photoreceptors and in the CNS and will provide potential targets for diagnostic or therapeutic strategies controling photoreceptor cell fate and the treatment of aging and neurological diseases. 

## Figures and Tables

**Table 1:** The table summarizes the principal effects produced by sphingolipids on PLA2, PLD, and LPPs enzymes.

	Sphingolipid effect	Ref.
PLA2	(i) Cer and C1P regulate eicosanoid synthesis through the activation of cPLA2*α* by favouring its transmembrane translocation and interaction with PtdCho.	[26, 39–42]
	(ii) Cer and SM influence sPLA2IIa fatty acid specificity by stimulating and inhibiting the release of C20:4 and C18:2, respectively.	[18, 37, 46]
	(iii) SM is a physiological inhibitor of sPLA2IIa, sPLA2V, and cPLA2.	[49–51]
	(iv) Cer and iPLA2*β* in association with mitochondria participate in endoplasmic reticulum stress-induced apoptosis.	[61, 64]

PLD	(i) Cer inhibits PLD activity by preventing its activation by PKCs and monomeric G proteins, by regulating its gene transcription or by direct effect on the catalytic core of the enzyme. Cer also abolishes the PtdOH/LysoPtdOH-stimulation of PLD.	[71–74]
	(ii) Sph and S1P regulate cellular proliferation by activation of PLD which stimulates DNA synthesis.	[75]

LPPs	(i) Sph inhibits the Mg^2+^-dependent phosphatidate phosphohydrolase and LPPs activities, increasing the accumulation of PA relative to DAG.	[76, 77, 80]
	(ii) Cer increases the specific activity of LPPs thus reducing the mitogenic activity of their substrates PthOH and S1P.	[71]
	(iii) LPPs modulate responses mediated by S1P or LysoPthOH by regulating their extracellular availability as ligands and by controlling the accumulation of bioactive lipid phosphates downstream of G-protein receptor activation.	[81]

## Abbreviations

2-AG: 2-arachidonoylglycerol
BLROS: Rod outer segments from light-adapted retinas
CNS: Central nervous system
Cer: Ceramide
C1P: Ceramide 1-phosphate
CC: Cerebral cortex
DAG: Diacylglycerol
DROS: Rod outer segments from dark-adapted retinas
DAGL: Diacylglycerol lipases
LPPs: Lipid phosphate phosphatases
MAGL: Monoacylglycerol lipases
MAG: Monoacylglycerol
PA: Phosphatidic acid
ROS: Rod outer segments
Sph: Sphingosine
S1P: Sphingosine 1-phosphate
Syn: Synaptosomes.
